# The Optimal Age of *Helicobacter pylori* Screen‐and‐Treat for Gastric Cancer Prevention in the United States

**DOI:** 10.1111/hel.70039

**Published:** 2025-05-06

**Authors:** Duco T. Mülder, James F. O'Mahony, Dianqin Sun, Luuk A. van Duuren, Rosita van den Puttelaar, Matthias Harlass, Weiran Han, Robert J. Huang, Manon C. W. Spaander, Uri Ladabaum, Iris Lansdorp‐Vogelaar

**Affiliations:** ^1^ Department of Public Health Erasmus Medical Center Rotterdam the Netherlands; ^2^ School of Economics University College Dublin Dublin Ireland; ^3^ Division of Gastroenterology and Hepatology Stanford University School of Medicine Stanford California USA; ^4^ Department of Gastroenterology & Hepatology Erasmus Medical Center Rotterdam the Netherlands

**Keywords:** decision modeling, *Helicobacter pylori*, screening, stomach cancer

## Abstract

**Background:**

Recent American College of Gastroenterology (ACG) guidelines recommend screening and eradicating *Helicobacter pylori* (*H. pylori*) in high‐risk racial groups to prevent gastric cancer (GC), but do not provide guidance on the age to screen. We aimed to determine the optimal age for *H. pylori* screen‐and‐treat.

**Materials and Methods:**

We developed a new microsimulation model, MISCAN‐gastric, which was calibrated to SEER incidence and clinical studies on the natural history of GC. One‐time screen‐and‐treat at ages 20–65 was compared to a no‐screening scenario in terms of cumulative incidence reduction, number needed‐to‐screen (NNS) and number needed‐to‐treat (NNT) to prevent one GC case. The NNS represents the number of individuals that require testing to prevent one GC case, while the NNT reflects the number requiring treatment. The optimal age was investigated for a high‐risk population subgroup (non‐Hispanic [NH] Black males) and compared to other subgroups.

**Results:**

Without screening, 332 noncardia GC cases occurred in a population of 100,000 NH Black males. *H. pylori* screen‐and‐treat reduced cumulative incidence by 43% when performed at age 20, but only by 5% when performed at age 65. The NNS was lowest at age 30 and increased markedly at older ages. The estimated NNS for test‐ages 20, 30, 40, and 65 were 645, 563, 769, and 5487, respectively. The NNT was lowest at the youngest age (261) and increased with age to 448 at age 40 and 3681 at age 65. The NNT and NNS were substantially higher in groups with lower GC risk: the optimal NNT was four times higher in NH White females compared to non‐Hispanic Black males.

**Conclusion:**

*H. pylori* screen‐and‐treat maximized population benefits when performed before age 40, emphasizing the need for early interventions. When performed at the optimal age, the benefits of *H. pylori* screen‐and‐treat may outweigh the harms for high‐risk racial groups.

**Abbreviations**
ACG: American College of Gastroenterology
CISNET: Cancer Intervention Surveillance Modeling Network
EU: European Union
GC: gastric cancer

*H. . pylori*

:
*Helicobacter pylori*

MISCAN: Microsimulation Screening Analysis
NCI: National Cancer Institute
NH: non‐Hispanic
NHANES: National Health and Nutrition Examination Survey
SEER: Surveillance, Epidemiology, and End Results

## 1 Introduction

Systematic trial evidence from high‐risk countries has demonstrated that eradicating *Helicobacter pylori* ( *H. pylori* ) in asymptomatic populations is effective in preventing gastric cancer (GC) [1, 2]. These findings have prompted the implementation of *H. pylori* screen‐and‐treat in high‐risk populations like Taiwan and Japan [3, 4]. Recently, the American College of Gastroenterology (ACG) issued new guidelines recommending *H. pylori* screen‐and‐treat for high‐risk racial groups in the United States [5]. Similarly, the European Union (EU) has recommended screen‐and‐treat strategies in higher‐risk regions [6]. Neither of these guidelines specifies an age for screening. The optimal age to initiate *H. pylori* screen‐and‐treat remains unclear and depends on factors such as disease risk, the progression rate of precancerous lesions, and the impact of eradication on GC risk reduction. Clinical trials are unlikely to answer this question directly due to constraints regarding follow‐up times and sample sizes.

Microsimulation models are useful alternatives to trials in these situations, allowing integration of evidence from various sources such as data registries and clinical studies. Within the Cancer Intervention Surveillance Modeling Network (CISNET), such models have successfully informed prevention guidelines for multiple cancer types [7, 8, 9], but had not yet been developed for *H. pylori* and GC. This study is the initial presentation of a CISNET model for *H. pylori* and GC. This model was developed within the extensively validated Microsimulation Screening Analysis (MISCAN) framework [10] and is independently constructed alongside the other two CISNET models of Harvard‐Stanford and Columbia University [11].

The lack of age‐specific recommendations in current *H. pylori* guidelines may reduce the effectiveness of GC prevention if screen‐and‐treat is administered at suboptimal ages. To inform future iterations of guidelines [5], this study aims to investigate the optimal age of *H. pylori* screen‐and‐treat for high‐risk subgroups in the United States through decision modeling. The balance between burden and benefits is analyzed for non‐Hispanic (NH) Black people, a high‐risk population, and compared to lower‐risk NH White people, stratified by sex.

## 2 Materials and Methods

The MISCAN‐gastric model was developed to estimate the optimal age of *H. pylori* screen‐and‐treat in two racial groups: a high‐risk group (NH Black people) and a low‐risk group (NH White people). The model development process involved several key steps: defining the model structure, specifying model assumptions, and estimating the model parameters through calibration. The model structure and assumptions were developed in collaboration with clinical experts (M.S., U.L., R.H.) for biological plausibility. We then used the calibrated model to investigate the effect of *H. pylori* screen‐and‐treat on GC incidence by sex and race, and investigated whether the outcomes are sensitive to the obtained model parameters.

### 2.1 Model Structure

MISCAN‐gastric is a stochastic, semi‐Markov microsimulation model that can estimate the GC incidence and mortality under various intervention scenarios, such as endoscopic and *H. pylori* screening. It has been developed within the MISCAN framework (Figure S1) [10], which has been extensively validated and applied to guide policy for other cancer types [12, 13, 14]. The model simulates independent individual life histories from birth until death. Some individuals develop precursors of GC, which may eventually progress to GC. *H. pylori* infection affects an individual's development of GC and its precursors. In any state, individuals can die of other causes. By simulating the same individuals under scenarios with and without screening, the population‐level effects of screening can be estimated. MISCAN‐gastric is a time‐to‐event model: simulated individuals move between states from the time of one event (e.g., birth) to the next event (e.g., tumor onset). Events are discrete and mutually exclusive, and transitions are determined based on the dwell time in each state. MISCAN‐gastric was coded in Python 3.10. A complete specification of the mathematical structure and calibration of MISCAN‐gastric can be found in the model Appendix S1.

**FIGURE 1:**
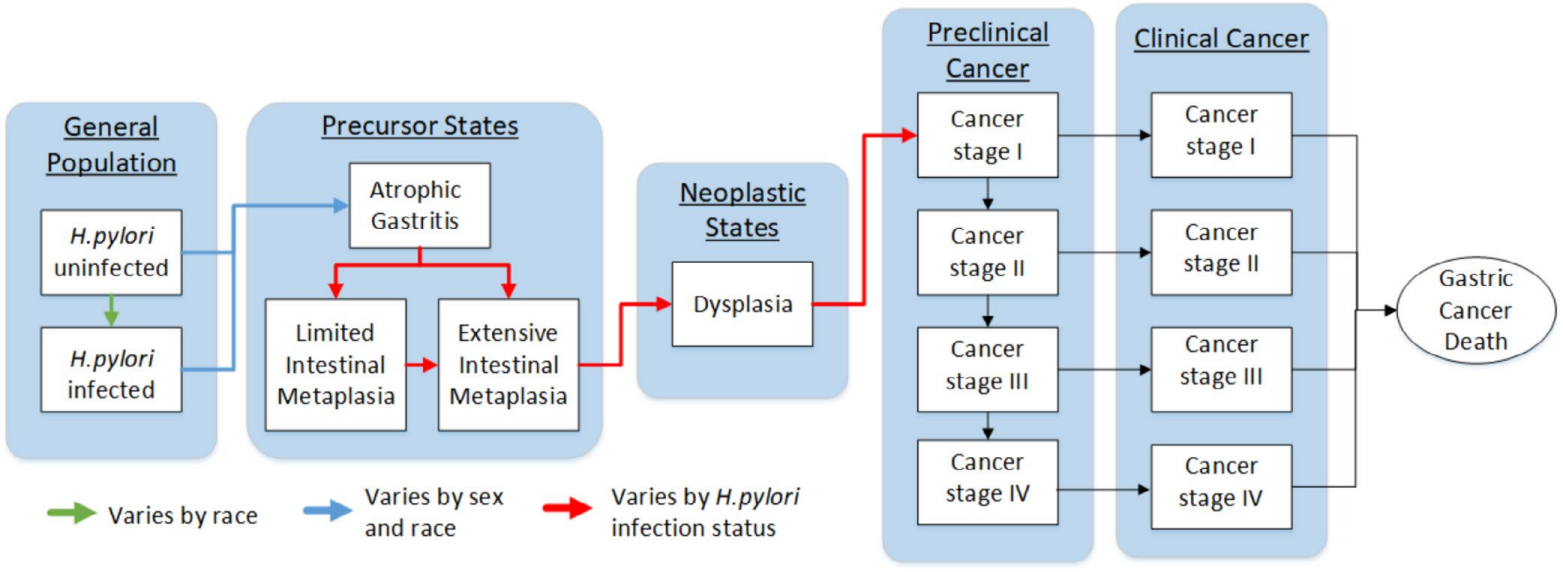
Natural history of the MISCAN‐gastric model. The arrows represent transitions between health states. The duration between transitions varies by race, sex and/or *H. pylori* infection status, depending on the color of the arrow. *H. pylori* , *Helicobacter pylori*.

MISCAN‐gastric's natural history model is based on Correa's cascade, encompassing the states of atrophic gastritis (AG), intestinal metaplasia (IM), dysplasia, and ultimately carcinoma (Figure 1) [15]. A distinction between limited (nonextensive) and extensive IM was incorporated to permit future assessment of surveillance strategies based on the extent of IM, which often features in clinical guidelines [16].

### 2.2 Model Assumptions

The model employs several assumptions, primarily due to insufficient data on precursor lesions in asymptomatic populations [17]. First, we assumed identical progression rates across racial groups. In contrast, the onset of precursor lesions was assumed to vary by race and sex. Second, *H. pylori* could elevate GC risk through two mechanisms: through an increased likelihood of developing precursor lesions (onset) or an accelerated progression of these lesions. The calibration decided how much each contributed. Regardless of the specific mechanism, the effect was assumed to be identical for all sex‐ and racial subgroups. Third, race‐specific prevalence of *H. pylori* was based on estimates derived from literature and NHANES data [18]. Finally, we assumed the same stage distribution at clinical diagnosis across racial groups, consistent with SEER data.

### 2.3 Model Calibration

Model parameters were calibrated to match noncardia GC (International Classification of Diseases [ICD] codes 16.1–16.6) incidence data from SEER 18 (November 2020) and various literature sources identified by the CISNET‐gastric group for a cohort of individuals born in 1940. For tumors with overlapping (ICD 16.8) and not‐otherwise‐specified (ICD 16.9) locations, we assumed the same proportion of noncardia to cardia cancers as in the other codes. Nonadenocarcinomas, including lymphomas and neuroendocrine tumors, were excluded, as these relatively rare cancers are recognized to have distinct natural histories and risk factors [19]. Furthermore, parameters on disease onset, progression, and differences by *H. pylori* status were calibrated to match clinical data on the impact of *H. pylori* on cancer risk [17, 20], precursor prevalence [17, 21, 22] and mean sojourn time [23] (Table 1).

**TABLE 1:** Data Used in the Calibration of MISCAN‐Gastric.

Data used in calibration	Value (95% CI)		Reference
Total mean sojourn time (time between onset of preclinical cancer stage I and clinical diagnosis)	3.7 (1.96–8.28 years)		[23]
OR of non‐cardia GC with *H. pylori* infection	4.79 (2.39–9.60)		[20]
Prevalence of *H. pylori* at age 35	White people: 36% Black people: 60%		[18]
Overall prevalence of atrophic gastritis	2.1% (0.7%–4.7%)		[17]
Overall prevalence of intestinal metaplasia	9.1% (6.9%–12.0%)		[17]
Overall prevalence of dysplasia	0.2% (0.04%–1.5%)		[24]
Odds ratio of developing precursor lesions of *H. pylori* + compared to *H. pylori* −	2.6 (1.5–3.3)		[17]
Odds ratios of intestinal metaplasia per age group	≤ 30 31–45 46–60 61–75 > 75	Ref. 1.7 2.7 3.9 5.3	[21]
Odds ratios of developing precursor lesions for males compared to females	1.04		[21]
Proportion of extensive cases of all intestinal metaplasia cases	28%		[25]
Relative risk of intestinal metaplasia progression to subsequent precursors after *H. pylori* eradication	0.8		[26]
Relative risk of atrophic gastritis progression to cancer following *H. pylori* eradication	0.28		[27]

Abbreviations: CI, confidence interval; GC, gastric cancer; *H. pylori* , *Helicobacter pylori* ; ref., reference.

### 2.4 *H. pylori* Screen‐and‐Treat Characteristics

We evaluated the impact of a single *H. pylori* screen‐and‐treat intervention. A cohort of 10 million individuals born in 1940 was simulated per subgroup. Screening involved a one‐time *H. pylori* test with 91.5% sensitivity, reflecting the performance of ^13^C‐urea breath tests (^13^C‐UBTs) [28]. We assumed a lifelong treatment success in 80% of treated individuals, aligning with success rates in the United States [29]. Unsuccessfully treated individuals remained infected. The reduction in GC risk following successful *H. pylori* eradication depended on the preneoplastic status at the time of eradication. If eradicated before precursors developed, *H. pylori* induced onset of precursors was fully prevented. If eradicated after precursor onset, progression was delayed, which was calibrated to match clinical findings [26, 27].

### 2.5 Analysis

We simulated 10 screening scenarios with one‐time *H. pylori* screen‐and‐treat at ages 20–65 in 5‐year intervals (20, 25, 30, …, 65). The effect of *H. pylori* screen‐and‐treat was determined by comparing GC incidence between scenarios with and without screening. The reduction in cumulative incidence, number needed‐to‐screen (NNS) and number needed‐to‐treat (NNT) for *H. pylori* to prevent one case of GC were evaluated for each screening scenario. The NNS refers to the number of individuals that require *H. pylori* testing to prevent one GC case. Similarly, the NNT refers to the number of screen‐positive individuals that require *H. pylori* treatment to prevent one GC case. Results were stratified by race and sex.

### 2.6 Sensitivity Analysis

Sensitivity analyses were conducted on (1) the calibrated natural history and (2) the characteristics of *H. pylori* screen‐and‐treat. For the first, sensitivity to the precursor dwell time was tested by recalibrating the onset of precursor disease while keeping the precursor dwell time parameters fixed at 70%–130% of the originally calibrated value in the base case. Additionally, the sensitivity to the effect of *H. pylori* on precursor dwell time was tested by assuming a faster progression in *H. pylori* ‐positive individuals (70%–90% faster progression rate than the originally calibrated value). Sensitivity to *H. pylori* infection age was tested by recalibrating the model while forcing a maximum infection age (age 5 and 20). For the second, sensitivity analyses on *H. pylori* screen‐and‐treat characteristics were performed by varying the test sensitivity, treatment success rate, and the effect of *H. pylori* on precursor progression. We compared the results for the age at which the NNT was lowest, the corresponding NNT, and the reduction in cumulative GC incidence.

## 3 Results

### 3.1 Calibration of the Natural History

**FIGURE 2:**
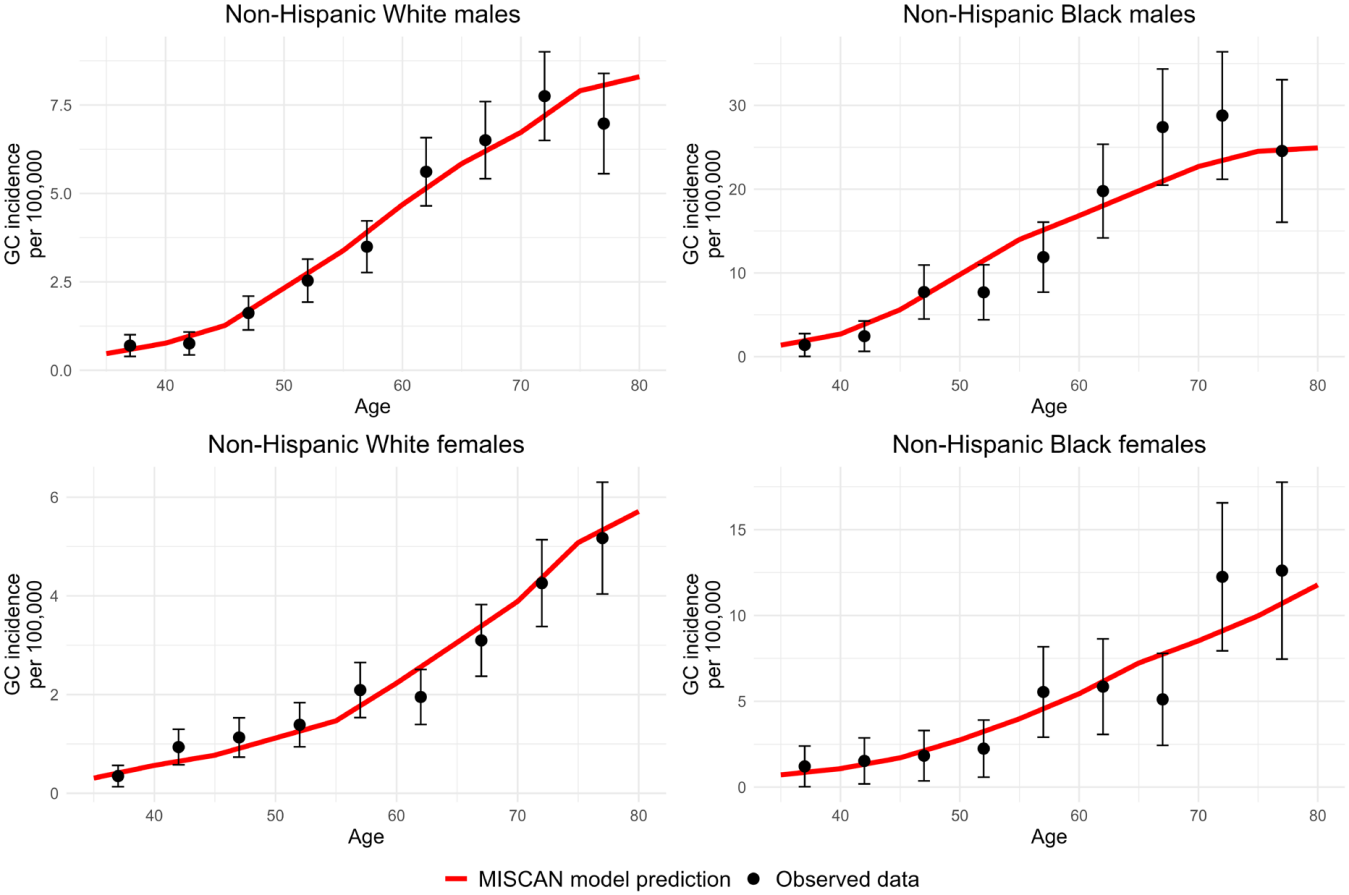
Calibration plot of gastric cancer incidence, specific to sex and race. GC, gastric cancer.

MISCAN‐gastric achieved a strong fit of the cancer incidence data across all population subgroups (Figure 2). Similarly, the fit on precursor prevalence was generally good, with varying goodness‐of‐fit across subgroups (Figures S2–S4). Following age patterns in cancer incidence, AG prevalence increased until age 55 in males and until 65 in females, followed by a decrease across all subgroups as AG progresses to IM. MISCAN‐gastric also accurately matched the observed stage distribution at clinical diagnosis (Table S3) and the calibration targets on the effect of *H. pylori* eradication on precursor progression (Table S4).

The estimated proportion of cancer attributable to *H. pylori* was 84% in the Black population compared to 67% in the White population, with no differences observed between sexes. While *H. pylori* increased the hazard of developing precursor lesions by 3.6, the impact on precursor progression was limited: progression was only 5% faster for AG and showed no increase for IM and dysplasia. Since the observed number of cancer cases is relatively low compared to the prevalence of AG and IM, progression rates of these precursors are also low. The proportions of people who developed clinical cancer out of those who ever had a precursor were 1.7% for AG and 2.0% for IM. This increased substantially for dysplasia: 48.7% of those who ever developed dysplasia also developed clinical cancer. Of the simulated individuals who developed clinical cancer, the average durations per health state for AG, IM, and dysplasia were 3.5, 17.9, and 6.4 years, respectively.

### 3.2 The Impact of *H. pylori* Screen‐And‐Treat on GC Incidence

**FIGURE 3:**
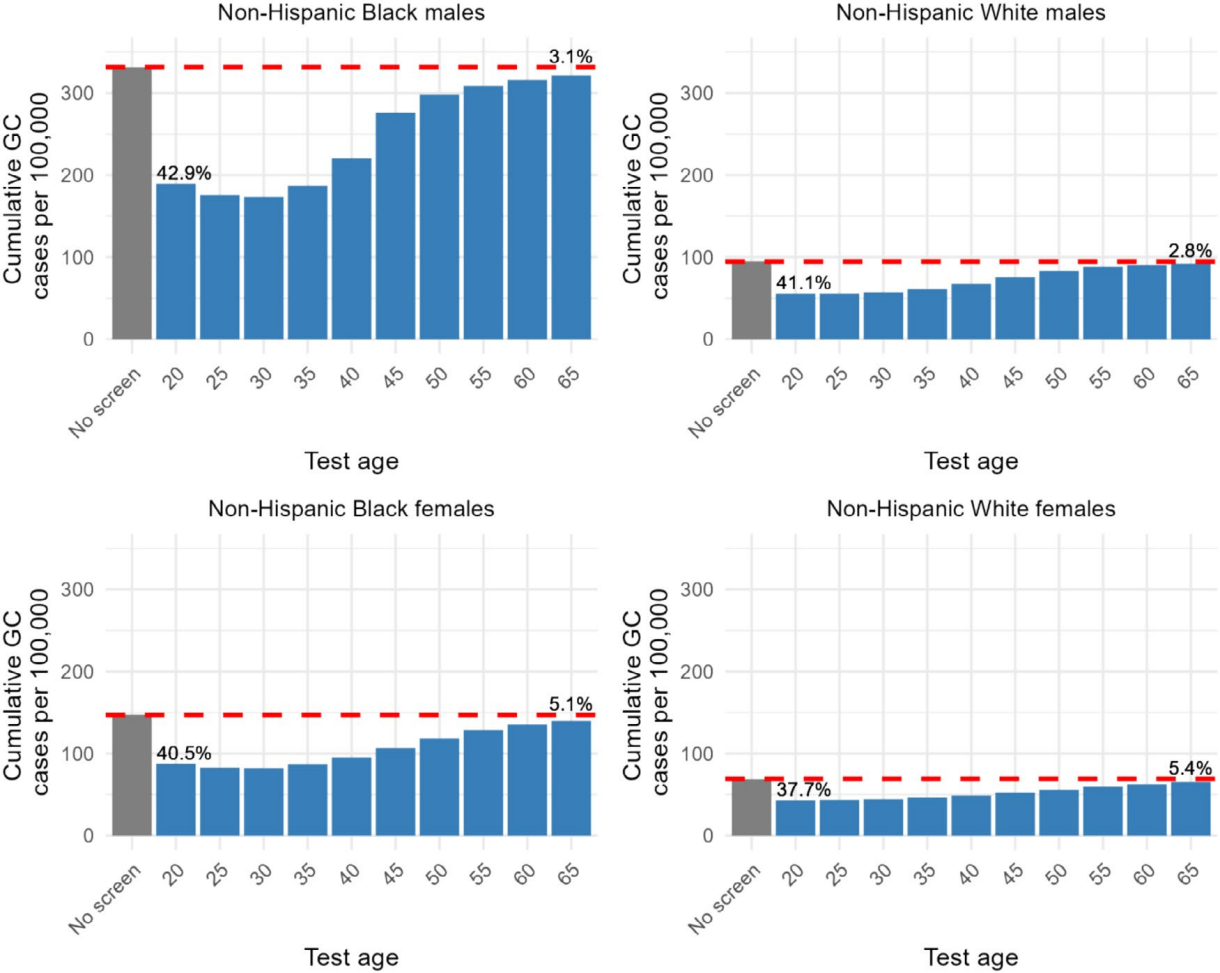
The cumulative incidence of gastric cancer per 100,000 in the absence of screening (gray bar) compared to the cumulative incidence under various one‐time *H. pylori* screen‐and‐treat scenarios performed at different ages (blue bars). The percentage in cumulative incidence reduction is shown for ages 20 and 65 in text.

**FIGURE 4:**
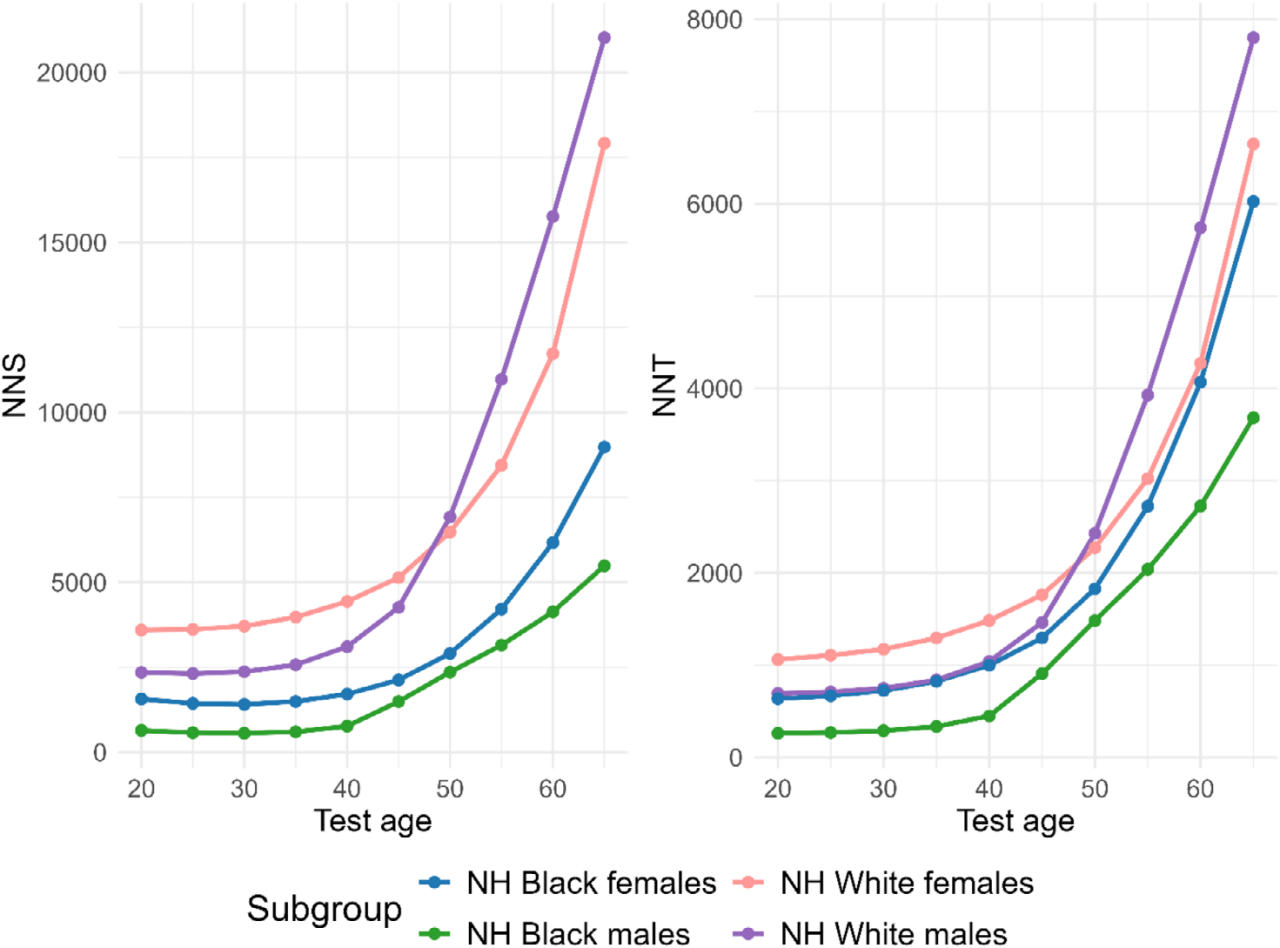
The number needed‐to‐screen (NNS) and number needed‐to‐treat (NNT) for *Helicobacter pylori* to prevent one case of gastric cancer. NH, non‐Hispanic.

Without screening, 332 noncardia GC cases occurred in a population of 100,000 NH Black males. In that group, *H. pylori* screen‐and‐treat was estimated to reduce GC incidence up to 47.6% when performed at age 30. The preventive effect decreased strongly with an increase in test age (Figure 3): only 3.1% of GC cases were prevented when screen‐and‐treat was performed at age 65. Consequently, optimal NNS and NNT were also achieved at younger test ages and increased with age. For screening at age 30, the NNS and NNT were 563 and 288, respectively (Figure 4, Table S5).

GC risk was considerably lower in other population subgroups, resulting in higher estimates of the NNT and NNS in these groups. Although different in absolute values, similar patterns were observed in the effect of screen‐and‐treat by test age, with a decline in preventive effect if tested at older ages (Figures 3 and 4). For NH Black females, NH White males, and NH White females, the NNTs were 723, 748, and 1171, respectively, while the corresponding NNS were 1411, 2372, and 3712.

### 3.3 Sensitivity Analyses

Like the base‐case analysis, *H. pylori* screen‐and‐treat achieved the lowest NNT at age 20 in all sensitivity analyses to both natural history and test parameters (Figure S5, Tables S6 and S7). The corresponding NNT remained stable despite changes in the calibrated natural history parameters, assumed test sensitivity, and the effect of eradication on precursor progression. However, the NNT was sensitive to changes in the assumed eradication rate, with a lower NNT observed in scenarios in which higher eradication rates were assumed (Table 2).

**TABLE 2:** Sensitivity Analyses of the Number Needed‐to‐Treat (NNT) to Prevent One Case of Gastric Cancer.

		Parameter value in sensitivity analysis	Number needed‐to‐treat at test‐age 20			
			NH Black males	NH White males	NH Black females	NH White females
	Base case	—	261	693	634	1060
Natural history parameters	Overall precursor dwell time	70%	243	659	592	1030
		80%	251	640	655	1024
		90%	240	655	542	990
		110%	229	599	595	1097
		120%	235	674	512	961
		130%	248	640	526	1062
	Effect of *H. pylori* infection on precursor progression	70%	229	636	578	971
		80%	225	613	559	936
		90%	222	595	542	906
	Maximum age of *H. pylori* infection	20	259	689	630	1050
		5	259	691	633	1056
Screen‐and‐treat parameters	Sensitivity (base case: 91.5%)	100%	264	707	646	1081
		80%	266	703	646	1067
		70%	267	702	645	1059
	Eradication rate (base case: 80%)	100%	213	566	518	860
		90%	236	630	573	955
		70%	303	805	738	1228
		60%	354	944	870	1441
	Effect of *H. pylori* eradication on AG dwell time (base case: ×3.6)	no effect	268	716	657	1102
		150%	265	708	646	1083
	Effect of *H. pylori* eradication on IM dwell time (base case ×1.1)	no effect	267	716	655	1100
		150%	262	699	631	1042

*Note:* The lowest NNT was achieved at test age 20 in all sensitivity analyses. For the sensitivity analyses on the natural history parameters, the model was recalibrated while fixing a subset of parameters at a different value than the calibrated solutions.

## 4 Discussion

This study is the first presentation of a CISNET model of GC and investigates the optimal age of *H. pylori* screen‐and‐treat in the United States, specifically for high‐risk subpopulations. Our modeling demonstrates that *H. pylori* screen‐and‐treat in the Black male population could prevent up to 48% of the GC cases if conducted at age 30. However, the potential population impact declines strongly as screening age rises, with only a 3% reduction in incidence observed when performed at age 65. Similarly, the lowest NNS and NNT were observed when screening at younger ages. Overall, the NNS and NNT were substantially worse in groups with lower GC risk. For example, the NNT was at least four times higher in the White female (lowest‐risk group) compared to the Black male (highest‐risk group) population.

Differences in the impact of *H. pylori* screen‐and‐treat on GC incidence by age are reflected in clinical data on the natural history of GC and the role of eradication in preventing disease progression. By integrating these data through microsimulation modeling, we were able to quantify and better understand the dynamics of gastric carcinogenesis. Calibration to these data revealed that the modeled progression through the full Correa's cascade—from the onset of AG to clinical cancer diagnosis—takes 31 years on average. With GC incidence peaking around age 70, many patients have likely developed precursor lesions by age 40. Because the effectiveness of *H. pylori* eradication in preventing disease progression was calibrated to decline as precursor lesions become more severe, the population‐level impact of screen‐and‐treat decreases substantially after age 40. As estimated mortality reductions were not sensitive to variations in the effect of eradication on precursor progression, it is likely that the greatest benefit of *H. pylori* eradication lies in preventing the onset of precursor lesions, rather than inhibiting their progression.

A decrease in effectiveness of *H. pylori* screen‐and‐treat by age is consistent with clinical findings from Asia and the Maastricht/Florence guidelines [30]. A recent Chinese randomized trial demonstrated that *H. pylori* eradication reduced GC incidence by 35% when performed before age 45 [31]. At test ages 20–45, our model predicted comparable reductions in incidence ranging from 29%–48%, depending on the exact test age and population subgroup. Consistent with MISCAN‐gastric, the trial found no significant population benefit of *H. pylori* screen‐and‐treat at older ages. Although conducted in high‐risk settings, other modeling studies further support our conclusion that *H. pylori* screen‐and‐treat is most effective at young ages [32, 33, 34, 35]. However, this finding should be interpreted with caution. Aligning with findings from a population‐based study in Hong Kong, our model indicated that eradication at older ages still reduced GC risk [36]. Individual patients at higher ages may therefore still benefit from *H. pylori* eradication, even though population benefits are maximized when screen‐and‐treat is performed before age 40. Further clinical studies are needed to corroborate our findings and refine strategies for implementing *H. pylori* screen‐and‐treat in populations with diverse risk levels.

Our findings carry important implications for effective GC prevention policies. Recent guidelines from the ACG and the EU recommended a screen‐and‐treat approach for high‐risk populations, but did not suggest the age for the intervention [5, 6]. Our study highlights the critical role of the test age in balancing the harms–benefits of *H. pylori* screen‐and‐treat strategies for high‐risk groups. While the NNS for NH Black males at test age 30 (NNS: 563) is lower than that for stool‐based screening tests for colorectal cancer [37], which are recommended for all sexes and racial groups, the NNS increases strongly if the test is performed later in life, reaching 5487 at age 65. These findings underscore the importance of early interventions to maximize benefits in the NH Black population and other racial groups with similar risk profiles, including Hispanic, Asian, and Indigenous American populations. Implementation of such race‐based interventions should be accompanied by careful consideration of ethical concerns, particularly regarding stigmatization and misclassification, to ensure equitable and responsible public health practices [38].

This study should be seen as a first exploration rather than the final verdict on *H. pylori* screen‐and‐treat strategies in the United States. Additional (modeling) studies are essential to inform future versions of guidelines, such as those recommended by the ACG [5]. First, a comprehensive cost‐effectiveness analysis is required before making conclusive recommendations. This analysis should evaluate the benefits of screening at a younger age in terms of efficacy, while also considering potential drawbacks, such as the increased number of tests that may be needed and the longer time frame required to see demonstrated benefits. Moreover, such analysis should take a broader perspective beyond GC and could include the prevention of other *H. pylori* ‐associated diseases, such as peptic ulcer disease, alongside consideration of potential harms related to increased antibiotic resistance. Second, since neither ACG nor EU guidelines define what constitutes high‐risk [5, 6], modeling studies could play a crucial role in identifying the thresholds at which the benefits of screen‐and‐treat outweigh the harms. Finally, modeling studies could aid in optimizing screening strategies, including determining the most effective test, the ideal number of repeat tests, and the potential role of upper endoscopy in prevention. These efforts would benefit from comparative modeling approaches, such as those initiated by CISNET.

While MISCAN‐gastric may provide a strong foundation for healthcare policy analysis, the quality of the model projections relies on the model assumptions. To inform these assumptions, our group collaborated on two systematic reviews examining the prevalence and progression rate of GC precursor lesions [17, 39]. These reviews demonstrated that differences in GC incidence are driven by variations in precursor onset, rather than differences in precursor progression rates. Based on those reviews, we assumed similar progression rates, but varying onsets of precursor lesions across population subgroups. We also had to make assumptions about *H. pylori* prevalence, as key details—such as the age of infection—remained uncertain despite the availability of reviews and NHANES data [18]. Nevertheless, sensitivity analyses indicated that variations in these assumptions had little impact on the optimal age of screen‐and‐treat, demonstrating the robustness of our findings. Notably, the NNT was sensitive to the eradication rate, stressing the importance of effective *H. pylori* treatments and close monitoring of antibiotic drug resistance following mass prescription of antibiotics. The development within the CISNET‐consortium, integration into the extensively validated MISCAN framework, and collaboration with clinical experts further strengthen our confidence in our model's estimates.

Our analysis has several limitations. First, we assumed a relatively simple natural history model based on Correa's cascade, which does not explicitly account for the regression of precursor lesions or risk factors beyond *H. pylori* . Incorporating additional factors, such as smoking and salt intake, may improve the granularity of the results. Nevertheless, the model's strong calibration fit suggests that its current structure adequately reflects clinical practice. Second, our model only includes two racial groups; future analyses could incorporate additional groups, particularly those at elevated risk. Third, we did not account for spill‐over preventive effects of *H. pylori* eradication, wherein treated individuals no longer transmit the infection. Given the high rate of intrafamilial *H. pylori* transmission at young ages [40], this may particularly underestimate the benefits of screen‐and‐treat at ages before parenthood. Including this effect would likely strengthen our conclusion that screen‐and‐treat is most effective at younger ages. Finally, MISCAN‐gastric would benefit from further external validation against trials and formal comparison with other GC models such as those developed within CISNET.

## 5 Conclusion

*H. pylori* screen‐and‐treat in high‐risk subpopulations in the United States maximized population benefits when performed before the age of 40. The efficacy declines sharply beyond this age, as a larger proportion of individuals has developed precursor lesions by then, reducing the impact of eradication on disease progression. This finding emphasizes the importance of early interventions to maximize benefits. While the relative effect of eradication was similar across population subgroups, the absolute effect varied significantly due to differences in GC risk. Even in low‐risk countries, the benefits of *H. pylori* screen‐and‐treat may therefore outweigh the harms for groups at high risk, when performed at younger ages.

## Author Contributions

**Duco T. Mülder:** conceptualization, methodology, formal analysis, investigation, writing – original draft, visualization. **James F. O'Mahony:** supervision, methodology, formal analysis, writing – review and editing. **Dianqin Sun:** methodology, investigation, writing – review and editing. **Luuk A. van Duuren:** methodology, investigation, writing – review and editing. **Rosita van den Puttelaar:** methodology, investigation, writing – review and editing. **Matthias Harlass:** methodology, investigation, writing – review and editing. **Weiran Han:** methodology, investigation, writing – review and editing. **Robert J. Huang:** writing – review and editing. **Uri Ladabaum:** writing – review and editing. **Manon C. W. Spaander:** writing – review and editing. **Iris Lansdorp‐Vogelaar:** supervision, methodology, funding acquisition, writing – review and editing. All authors reviewed and approved the final manuscript.

## Disclosure

Disclaimer: The content is solely the responsibility of the authors and does not necessarily represent the official views of the National Institutes of Health.

## Conflicts of Interest

The authors declare no conflicts of interest.

## Supporting information


Appendix S1.

## Data Availability

The data underlying this article are available in the article and in its Tables S1 and S2.
