# Supplementary material for: The Optimal Age of Helicobacter pylori Screen‐and‐Treat for Gastric Cancer Prevention in the United States
Source: Helicobacter. 2025 May 6;30(3):e70039. doi: 10.1111/hel.70039 (PMC12056297; doi:10.1111/hel.70039)
Supplement: Supplementary file 1 — Appendix S1. [file HEL-30-e70039-s001.docx]

# Supplementary material

## Appendix part I: Model appendix of MISCAN-gastric

**Suppl. Figure 1:** The structure of the MISCAN framework

**Suppl. Table 1:** Data used in the calibration of MISCAN-gastric

**Sup. Table 2:** Calibrated values of the model parameters of MISCAN-gastric

**Suppl. Figure 2:** Calibration plot of the prevalence of atrophic gastritis, specific to sex and *Helicobacter pylori* infection status.

**Suppl. Figure 3:** Calibration plot of the prevalence of limited intestinal metaplasia, specific to sex and *Helicobacter pylori* infection status.

**Suppl. Figure 4:** Calibration plot of the prevalence of extensive intestinal metaplasia, specific to sex and *Helicobacter pylori* infection status.

**Suppl. Table 3:** Calibration fit of the stage-distribution at clinical detection as predicted by MISCAN-gastric vs. observed in SEER-18.

**Suppl. Table 4:** Calibration fit to the effect of *H. pylori* eradication.

**Suppl. Table 5:** The Number Needed to Treat (NNT) and Number Needed to Screen (NNS) for *Helicobacter pylori* to prevent one case of gastric cancer.

## Appendix part II: Sensitivity analyses

**Suppl. Figure 5:** Calibration plot of gastric cancer incidence, specific to sex and race of the sensitivity analyses.

**Suppl. Table 6.** Sensitivity analyses on the natural history parameters of MISCAN-gastric.

**Suppl. Table 7.** Sensitivity analyses on the screen-and-treat parameters of MISCAN-gastric.

## Appendix part I: Model appendix of MISCAN-gastric

The Microsimulation Screening Analysis (MISCAN) gastric model is developed within the Early Detection & Screening programme in the Department of Public Health at the Erasmus University Medical Center in Rotterdam, the Netherlands (1). It is a stochastic, semi-Markov microsimulation model that can estimate the effect of intervention strategies on GC incidence and mortality.

**MISCAN structure**

As MISCAN-gastric is a microsimulation model, the model simulates independent individual life histories from birth until death, rather than as proportions of a cohort. This structure is similar across all MISCAN models, such as MISCAN-colon (2). This allows future state transitions to depend on past transitions, giving individuals a memory function. Unlike most traditional Markov models, MISCAN-gastric does not use yearly/monthly transition probabilities. Instead, in each health state, individual durations to other health states are generated. The term stochastic implies that model uses probability distributions and durations to simulate events, rather than using fixed values. The results are therefore subject to random variation. In MISCAN-gastric, some individuals develop precursor lesions which may eventually progress to cancer.

**Suppl. Figure 1:** The structure of the MISCAN framework (1)

The MISCAN-framework consists of three modules: demography, natural history and screening (Figure 1) (1). This framework has been extensively validated and applied for guiding cancer policy for other types of cancer in various contexts (3-5). In the demography part, simulated individuals are born and die according to the population characteristics. The natural history part determines how many people develop precursor lesions and what proportion progresses to cancer. The screening part contains information about the screening test, such as the sensitivity and the effect of treatment. MISCAN models can be run with and without screening and prevention strategies. Comparing these scenarios can then inform the extent to which screening affects disease outcomes, such as incidence and mortality. With the goal of model transparency, this appendix focuses on the natural history assumptions of the MISCAN-gastric model.

**Natural history of MISCAN-gastric**

MISCAN-gastric’s natural history model is based on Correa’s cascade, encompassing the states of atrophic gastritis (AG), intestinal metaplasia (IM), dysplasia, and ultimately carcinoma (Main Figure 1) (6). A distinction between limited (non-extensive) and extensive IM was incorporated to permit future assessment of surveillance strategies based on the extent of IM, which often features in clinical guidelines (7).

Mathematically, the onset age of precursor lesions was based on an *H. pylori*-specific hazard and race- and sex-specific generalized logistic hazard functions (blue arrows in Main Figure 1). The dwell times of health states followed Weibull distributions (red and black arrows in Main Figure 1), similar to the approach in other cancer natural history models(8, 9). These transitions will now be further explained mathematically in the sequence of the model according to figure 2.

Atrophic gastritis onset risk

The onset age of precursor lesions was based on an *H. pylori*-specific hazard $H_{Hp}$ and a race- and sex-specific generalized logistic hazard functions. The hazard for individuals uninfected with *H. pylori* was set to one. The logistic hazard function was rewritten for interpretability. We define a constant:

$$b_{s,r}= \frac{log(\frac{1}{v_{s,r}}({\frac{K_{s,r}}{L})}^{v}-\frac{1}{v_{s,r}})}{G_{s,r}^{m}-M_{s,r}},$$

To define the onset, based on random number *x*:

$${Onset age}_{i}(x)= \frac{K_{s,r}}{{(1+v_{s,r}\times exp(-b_{s,r}\left( x-M_{s,r} \right)))}^{1/v_{s,r}}}.$$

Where *L* is a small number larger than 0 (0.001).

Calibrated parameters: $K_{s,r}, v_{s,r}$, $M_{s,r}$, $G_{s,r}^{m}$ for each sex *s* (male and female) and race *r* (black and white). x follows an exponential distribution with an *H. pylori* specific Hazard rate.

The parameters can be interpreted as follows:

- $K_{s,r}$ refers to the horizontal asymptote of the function.
- $v_{s,r}$, reflects the steepness in the inflection point.
- $M_{s,r}$ is the age of the inflection point
- $G_{s,r}^{m}$ is the age for which the function form equals L (0.001)

Atrophic gastritis progression:

Two independent dwell times are drawn from a Weibull distribution with varying means $k_{AG,1}$ and $k_{AG,2}$ but the same scale parameter $\lambda_{AG}$:

$${Dwell AG}_{1}\sim WB(k_{AG,1}* Ω_{AG}^{I\{Hp\}},\lambda_{AG})$$

$${Dwell AG}_{2}\sim WB\left( k_{AG,2}* Ω_{AG}^{I\left\{ Hp \right\}},\lambda_{AG} \right),$$

where $Ω_{AG}^{I\{Hp\}}$ represents the effect of *Helicobacter pylori (H. pylori*) on the mean progression time. $Ω_{AG}^{I\{Hp\}}$ is a calibrated factor between 0 and 1, depending on *H. pylori* infection.

If ${AG}_{1} \leq{AG}_{2}$, the individual progresses to limited intestinal metaplasia (IM). Otherwise, they progress to extensive IM directly.

Calibration parameters: $k_{AG,1}$, $k_{AG,2}$, $\lambda_{AG}$, $Ω_{AG}^{I\{Hp\}}$

IM progression:

For limited IM, a dwell time to extensive IM is drawn from a Weibull distribution:

$$Dwell limited IM \sim WB(k_{IM lim}* Ω_{IM/dys}^{I\{Hp\}},\lambda_{IM})$$

For extensive IM, we draw a dwell time to dysplasia with a different mean, but the same scale parameter:

$$Dwell extensive IM\sim WB(k_{IM ext}* Ω_{IM/dys}^{I\{Hp\}},\lambda_{IM})$$

The parameter $Ω_{IM/dys}^{I\{Hp\}}$ again represents the effect of *H. pylori* on the mean dwell time. Note that this effect is the same for IM and dysplasia.

To account for the effect of age on progression of disease, these dwell times are multiplied by a factor:

$$1+ \rho*age$$

Where age is the midpoint between the onset age of IM and the next state.

Calibration parameters: $k_{IM lim}$, $k_{IM ext}$, $\lambda_{IM}$, $Ω_{IM/dys}^{I\{Hp\}}$, $\rho$

Dysplasia progression:

For dysplasia, we draw a dwell time to preclinical cancer stage 1 from a Weibull distribution:

$$Dwell dysplasia\sim WB(k_{dys}* Ω_{IM/dys}^{I\{Hp\}},\lambda_{dys})$$

Again, we account for the effect of age by multiplying the dwell times by$1+ \rho*age$, which is the same factor used in the dwell time of IM.

Additional calibration parameters:$k_{dys}$,$\lambda_{dys}$.

Preclinical cancer progression

In each preclinical cancer stage j, we draw a dwell time to preclinical stage j+1 from an exponential distribution:

$$Dwell cancer S_{j, j+1}\sim\exp\left( \lambda_{j} \right)$$

For j=1,2,3.

In each preclinical cancer stage, we also draw a dwell time until clinical detection:

$$Time until detection cancer S_{j,}\sim\exp\left( \alpha_{j} \right)$$

For j=1,2,3,4

Preclinical cancers progress to the next stage if:

$$Dwell cancer S_{j, j+1}\leq Time until detection cancer S_{j,}$$

Otherwise, cancers are clinically detected after the time until detection has elapsed.

Calibration parameters: $\lambda_{1}, \lambda_{2,}\lambda_{3}$ and $\alpha_{1}$, $\alpha_{2}$, $\alpha_{3}$,$\alpha_{4}$

**Calibration**

The model was calibrated to SEER incidence data and data from studies. An overview of the data used from clinical studies can be found in Suppl. Table 1. The model’s goodness-of-fit was quantified using binomial, multinomial and Poisson deviance functions for proportions (prevalence metrics), fractions and (incidence) rates, respectively (10). The sum of deviances was minimized through a genetic optimization algorithm (11). Latin Hypercube Sampling was used to select the initial parameter-set (12). Calibration and model runs used 10 million individuals per simulation. An overview of the calibrated model parameters can be found in Suppl. table 2. Calibration fit is demonstrated in Main Figure 2, Suppl. Figures 2-4 and Suppl. Tables 3 and 4.

**Suppl. Table 1:** Data used in the calibration of MISCAN-gastric

| Data used in calibration | Value (95% CI) | | Reference |
| --- | --- | --- | --- |
| Total mean sojourn time (time between onset of preclinical cancer stage I and clinical diagnosis) | 3.7 (1.96-8.28 years) | | (13) |
| OR of non-cardia GC with *H. pylori* infection | 4.79 (2.39-9.60) | | (14) |
| Prevalence of *H. pylori* at age 35 | White people: 36%  Black people: 60% | | (15) |
| Overall prevalence of atrophic gastritis | 2.1% (0.7-4.7%) | | (16) |
| Overall prevalence of intestinal metaplasia | 9.1% (6.9-12.0%) | | (16) |
| Overall prevalence of dysplasia | 0.2% (0.04%-1.5%) | | (17) |
| Odds ratio of developing precursor lesions of *H. pylori+* compared to *H. pylori*- | 2.6 (1.5-3.3) | | (16) |
| Odds ratios of intestinal metaplasia per age group | ≤30  31-45  46-60  61-75  >75 | Ref.  1.7  2.7  3.9  5.3 | (18) |
| Odds ratios of developing precursor lesions for males compared to females | 1.04 | | (18) |
| Proportion of extensive cases of all intestinal metaplasia cases | 28% | | (19) |
| Relative risk of intestinal metaplasia progression to subsequent precursors after *H. pylori* eradication | 0.8 | | (20) |
| Relative risk of intestinal metaplasia progression to cancer following *H. pylori* eradication | 0.7 | | (21) |
| Relative risk of atrophic gastritis progression to cancer following *H. pylori* eradication | 0.28 | | (21) |

**Sup. Table 2:** Calibrated values of the model parameters of MISCAN-gastric

| Parameters | Interpretation | Calibrated value | |
| --- | --- | --- | --- |
| $\boldsymbol{H}_{\boldsymbol{Hp}}$ | Hazard rate of developing atrophic gastritis for *H. pylori* infected individuals. | 3.651151191 | |
| $\boldsymbol{K}_{\boldsymbol{s,r}}$ | Horizontal asymptote o of the onset age function | Black males  White males  Black females  White females | 0.135548  0.062793  0.080278  0.083563 |
| $\boldsymbol{v}_{\boldsymbol{s,r}}$, | Inflection point of the onset age function | Black males  White males  Black females  White females | 19.54042  1.769942  1.006667  1.242386 |
| $\boldsymbol{M}_{\boldsymbol{s,r}}$ | Age of the inflection point of the onset age function | Black males  White males  Black females  White females | 44.65589  46.55290  52.38103  61.61536 |
| $\boldsymbol{G}_{\boldsymbol{s,r}}^{\boldsymbol{m}}$ | Age for which the onset age function equals 0.001 | Black males  White males  Black females  White females | 0.833748  8.716735  1.437170  0.833748 |
| $\boldsymbol{k}_{\boldsymbol{AG,1}}$ | Weibull mean dwell time for atrophic gastritis to limited IM | 23.43013009 | |
| $\boldsymbol{k}_{\boldsymbol{AG,2}}$ | Weibull mean dwell time for atrophic gastritis to extensive IM | 49.89100247 | |
| $\boldsymbol{Ω}_{\boldsymbol{AG}}^{\boldsymbol{I\{Hp\}}}$ | Effect of *H. pylori* infection on progression time of atrophic gastritis | 1 | |
| $\boldsymbol{\lambda}_{\boldsymbol{AG}}$ | Scale of the Weibull distributions of atrophic gastritis dwell time | 0.571882689 | |
| $\boldsymbol{k}_{\boldsymbol{IM lim}}$ | Weibull mean dwell time for limited IM to extensive IM | 74.07276355 | |
| $\boldsymbol{Ω}_{\boldsymbol{IM/dys}}^{\boldsymbol{I\{Hp\}}}$ | Effect of *H. pylori* infection on the dwell time of IM and dysplasia | 0.960589394 | |
| $\boldsymbol{\lambda}_{\boldsymbol{IM}}$ | Scale of the Weibull distributions of IM dwell time | 1.226024 | |
| $\boldsymbol{k}_{\boldsymbol{IM ext}}$ | Weibull mean dwell time for extensive IM to dysplasia | 37.00643102 | |
| $\boldsymbol{\rho}$ | Parameter to model the effect of age on dwell time | 7.980778226 | |
| $\boldsymbol{k}_{\boldsymbol{dys}}$ | Weibull mean dwell time for dysplasia to preclinical cancer stage 1 | 4.388887775 | |
| $\boldsymbol{\lambda}_{\boldsymbol{dys}}$ | Scale of the Weibull distribution of dysplasia dwell time | 0.661216 | |
| $\boldsymbol{\lambda}_{\boldsymbol{j}}$ | Exponential distribution mean dwell time for preclinical cancer stage j to j+1 | j=1  j=2  j=3 | 3.297144  0.794827  1.137446 |
| $\boldsymbol{\alpha}_{\boldsymbol{j}}$ | Exponential distribution mean dwell time for preclinical cancer to become clinically detected in stage j | j=1  j=2  j=3  j=4 | 8.087736  3.714227  4.306321  0.428254 |
| $\mathbf{T}_{\boldsymbol{Hp,AG}}$ | Multiplication factor of remaining atrophic gastritis dwell time after *H. pylori* eradication | 3.554993 | |
| $\mathbf{T}_{\boldsymbol{Hp,IM/dys}}$ | Multiplication factor of remaining IM and dysplasia dwell time after *H. pylori* eradication | 1.125454 | |


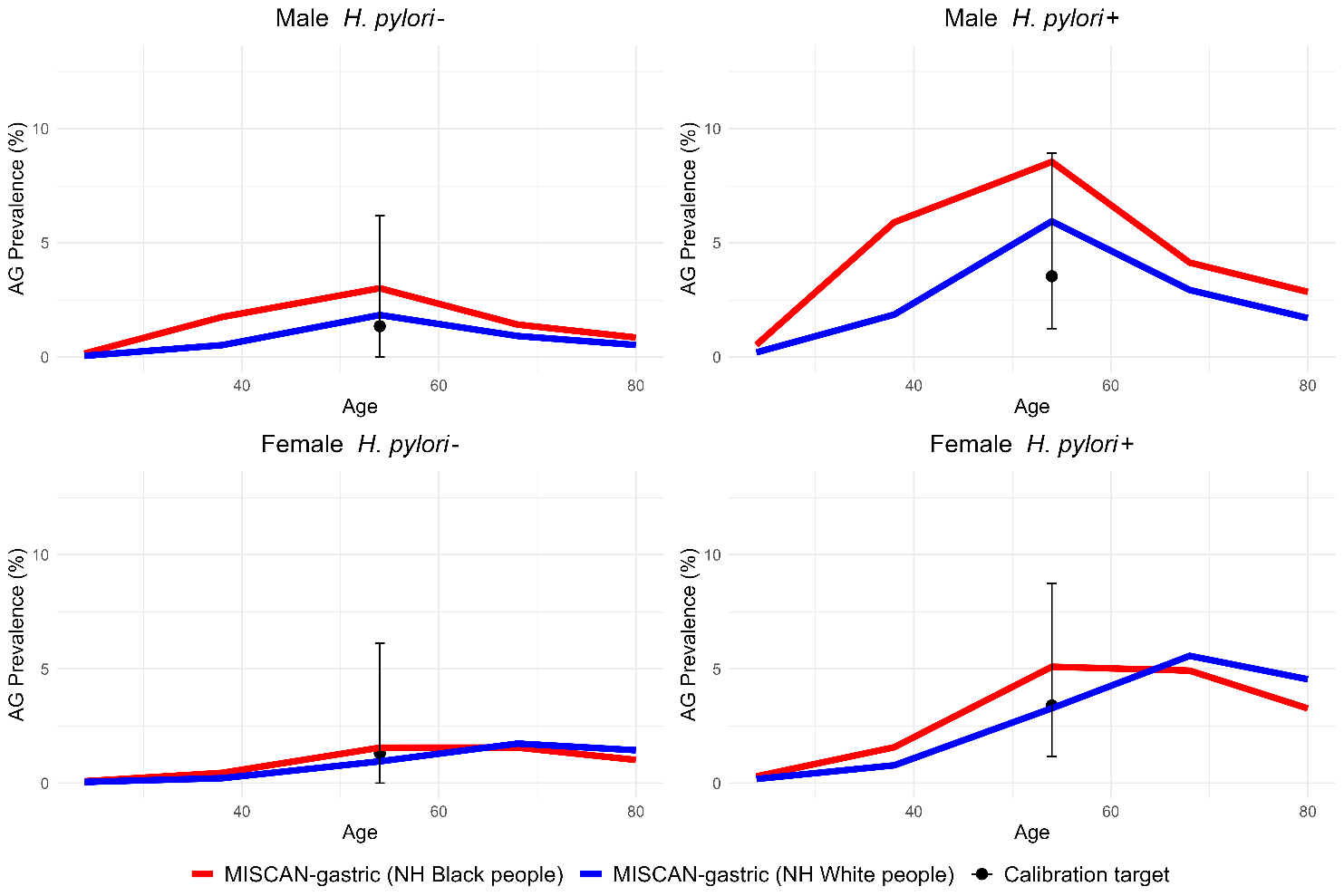


**Suppl. Figure 2:** Calibration plot of the prevalence of atrophic gastritis, specific to sex and *Helicobacter pylori* infection status. Note that the calibration targets consistent for Black and White people.

*H. pylori, Helicobacter pylori*; AG, atrophic gastritis; NH, non-Hispanic.


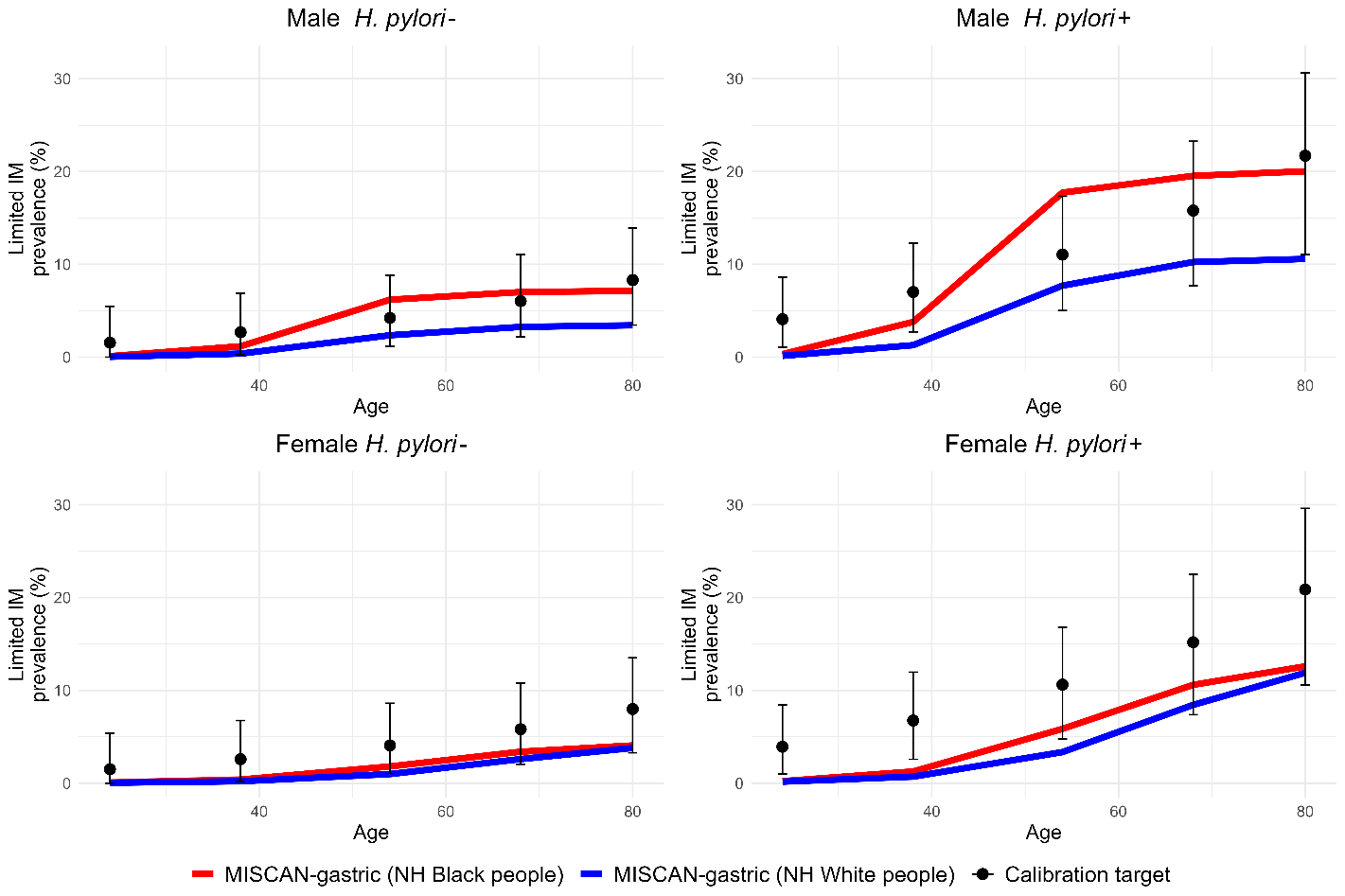


**Suppl. Figure 3:** Calibration plot of the prevalence of limited intestinal metaplasia, specific to sex and *Helicobacter pylori* infection status. Note that the calibration targets are consistent for Black and White people.

*H. pylori, Helicobacter pylori*; IM, intestinal metaplasia; NH, non-Hispanic.


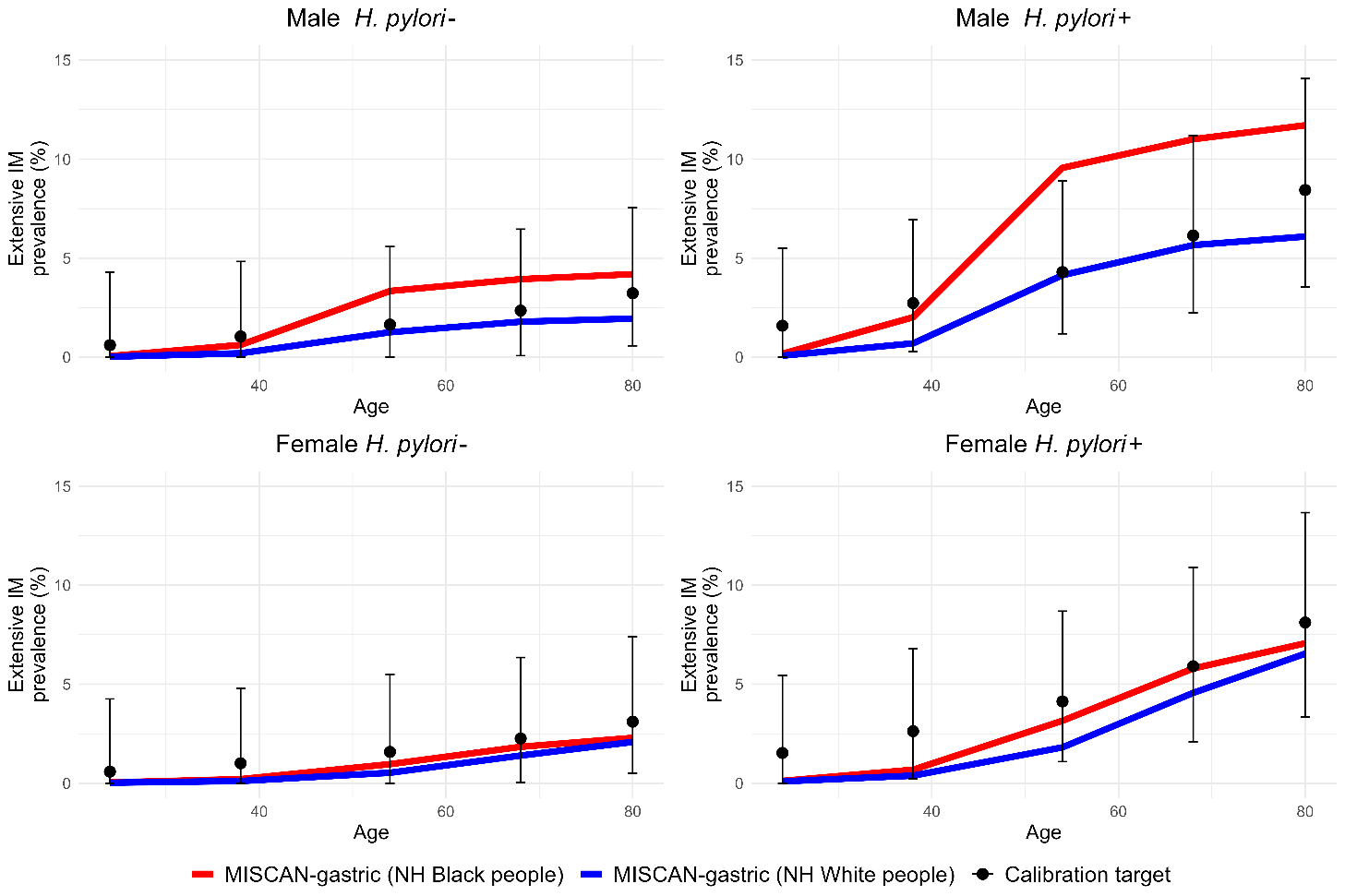


**Suppl. Figure 4:** Calibration plot of the prevalence of extensive intestinal metaplasia, specific to sex and *Helicobacter pylori* infection status. Note that the calibration targets are consistent for Black and White people.

*H. pylori, Helicobacter pylori*; IM, intestinal metaplasia; NH, non-Hispanic.

**Suppl. Table 3:** Calibration fit of the stage-distribution at clinical detection as predicted by MISCAN-gastric vs. observed in SEER-18.

| Stage | SEER 18 | MISCAN-gastric |
| --- | --- | --- |
| I | 28.8% | 30.2% |
| II | 12.7% | 12.9% |
| III | 12.5% | 11.8% |
| IV | 46.0% | 45.1% |

**Suppl. Table 4:** Calibration fit to the effect of *H. pylori* eradication. The target value is the relative risk of getting cancer after *H. pylori* eradication compared to those who did not receive eradication, as observed in clinical studies.

|  | Observed | MISCAN-gastric |
| --- | --- | --- |
| Atrophic gastritis | 0.28 (21) | 0.29 |
| Intestinal metaplasia | 0.8 (20) | 0.81 |

|  | Cumulative Incidence Reduction (%) | | | | Number Needed to Treat | | | | Number Needed to Screen | | | |
| --- | --- | --- | --- | --- | --- | --- | --- | --- | --- | --- | --- | --- |
| Test age | **NH Black males** | **NH White males** | **NH Black females** | **NH White females** | **NH Black males** | **NH White males** | **NH Black females** | **NH White females** | **NH Black males** | **NH White males** | **NH Black females** | **NH White females** |
| 20 | 42.9 | 41.1 | 40.5 | 37.7 | 261 | 693 | 634 | 1060 | 645 | 2352 | 1569 | 3600 |
| 25 | 47.0 | 41.1 | 43.7 | 37.1 | 268 | 709 | 666 | 1106 | 579 | 2319 | 1437 | 3617 |
| 30 | 47.6 | 39.6 | 44.1 | 35.8 | 288 | 748 | 723 | 1171 | 563 | 2372 | 1411 | 3712 |
| 35 | 43.5 | 35.8 | 41.0 | 33.0 | 333 | 838 | 822 | 1293 | 605 | 2576 | 1495 | 3977 |
| 40 | 33.4 | 29.0 | 35.2 | 29.0 | 448 | 1039 | 997 | 1483 | 769 | 3108 | 1713 | 4437 |
| 45 | 16.6 | 20.4 | 27.6 | 24.5 | 908 | 1461 | 1293 | 1761 | 1494 | 4264 | 2129 | 5140 |
| 50 | 10.0 | 12.0 | 19.6 | 18.8 | 1482 | 2428 | 1827 | 2272 | 2359 | 6923 | 2908 | 6478 |
| 55 | 7.0 | 7.0 | 12.8 | 13.7 | 2037 | 3927 | 2721 | 3022 | 3155 | 10971 | 4214 | 8443 |
| 60 | 4.8 | 4.4 | 8.2 | 9.2 | 2724 | 5741 | 4067 | 4273 | 4132 | 15761 | 6169 | 11726 |
| 65 | 3.1 | 2.8 | 5.1 | 5.4 | 3681 | 7803 | 6026 | 6650 | 5487 | 21022 | 8980 | 17917 |

**Suppl. Table 5:** The Number Needed to Treat (NNT) and Number Needed to Screen (NNS) for *Helicobacter pylori* to prevent one case of gastric cancer.

## Appendix part II: Sensitivity analyses


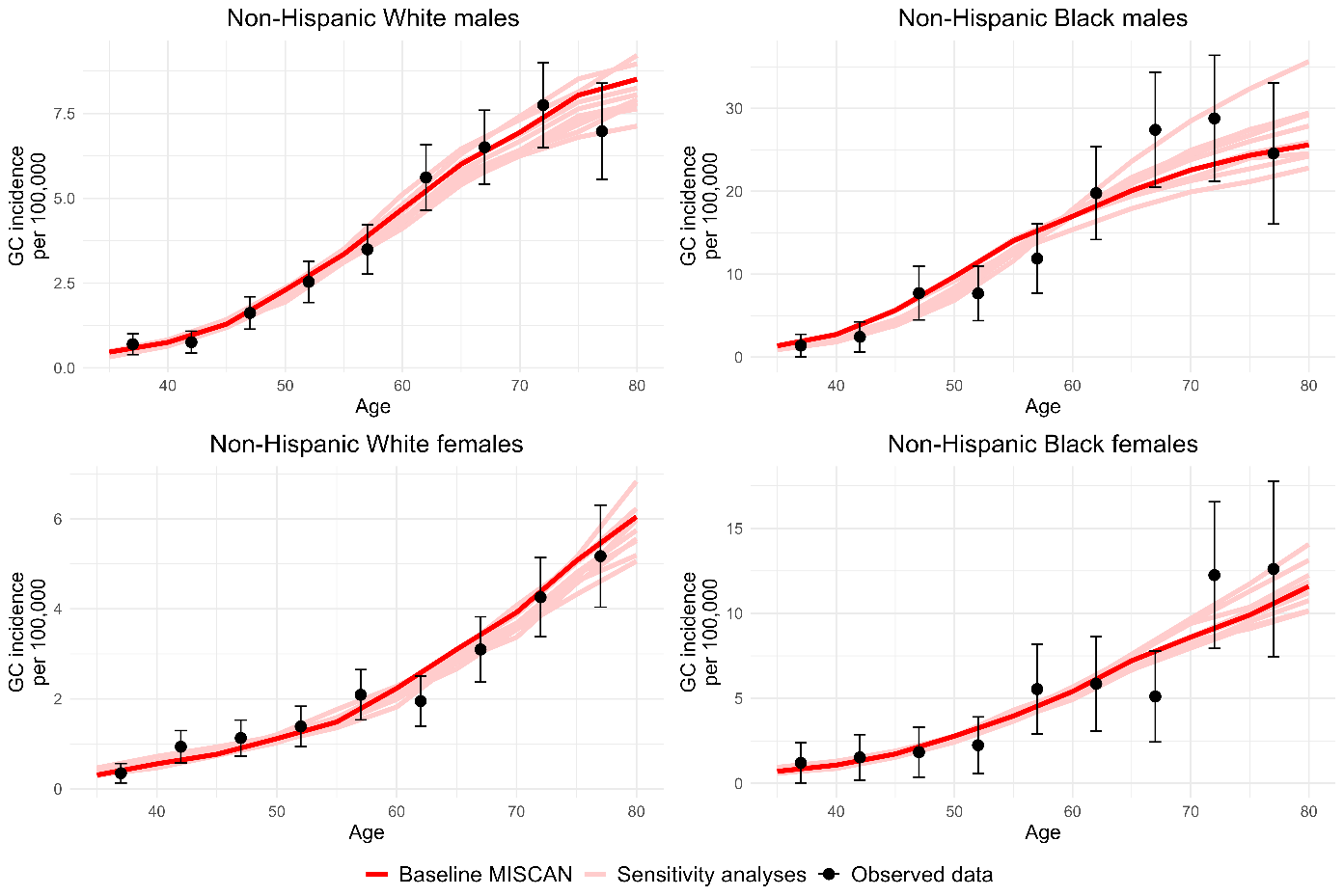


**Suppl. Figure 5:** Calibration plot of gastric cancer incidence, specific to sex and race of the sensitivity analyses. The red line indicates the fit of the baseline MISCAN gastric model. The light red lines indicate the fit of the recalibrated models for the sensitivity analyses. In these analyses, either the mean dwell time of precursor lesions or the effect of *Helicobacter pylori* on progression speed has been changed. The remaining parameters have been calibrated to fit cancer incidence. All recalibrated models also fit the target data.

GC, gastric cancer.

|  |  | Optimal age of test-and-treat | | | | Corresponding NNT | | | | Cumulative incidence reduction | | | |
| --- | --- | --- | --- | --- | --- | --- | --- | --- | --- | --- | --- | --- | --- |
|  | Parameter value compared to calibrated | NH Black males | NH White males | NH Black females | NH White females | NH Black males | NH White males | NH Black females | NH White females | NH Black males | NH White males | NH Black females | NH White females |
| Basecase |  | 20 | 20 | 20 | 20 | 261 | 693 | 634 | 1060 | 43% | 41% | 40% | 38% |
| Overall precursor dwell time | 70% | 20 | 20 | 20 | 20 | 243 | 659 | 592 | 1030 | 41% | 40% | 39% | 35% |
|  | 80% | 20 | 20 | 20 | 20 | 251 | 640 | 655 | 1024 | 42% | 41% | 39% | 36% |
|  | 90% | 20 | 20 | 20 | 20 | 240 | 655 | 542 | 990 | 43% | 42% | 41% | 37% |
|  | 110% | 20 | 20 | 20 | 20 | 229 | 599 | 595 | 1097 | 44% | 43% | 42% | 37% |
|  | 120% | 20 | 20 | 20 | 20 | 235 | 674 | 512 | 961 | 44% | 42% | 42% | 37% |
|  | 130% | 20 | 20 | 20 | 20 | 248 | 640 | 526 | 1062 | 43% | 42% | 42% | 37% |
| Effect of *H. pylori* infection on precursor progression | 70% | 20 | 20 | 20 | 20 | 229 | 636 | 578 | 971 | 43% | 42% | 41% | 39% |
|  | 80% | 20 | 20 | 20 | 20 | 225 | 613 | 559 | 936 | 44% | 43% | 41% | 39% |
|  | 90% | 20 | 20 | 20 | 20 | 222 | 595 | 542 | 906 | 44% | 43% | 42% | 40% |
| Maximum age of *H. pylori* infection | 20 | 20 | 20 | 20 | 20 | 259 | 689 | 630 | 1050 | 64% | 51% | 61% | 47% |
|  | 5 | 20 | 20 | 20 | 20 | 259 | 691 | 633 | 1056 | 64% | 51% | 60% | 46% |

**Suppl. Table 6.** Sensitivity analyses on the natural history parameters of MISCAN-gastric. For each analysis, the model was recalibrated while fixing a subset of parameters at a different value than the calibrated solutions. The optimal age to screen-and-treat (based on NNT) did not change in any analysis. NH, non-Hispanic; *H. pylori, Helicobacter pylori*.

|  |  | Optimal age of screen-and-treat | | | | Corresponding NNT | | | | Cumulative incidence reduction | | | |
| --- | --- | --- | --- | --- | --- | --- | --- | --- | --- | --- | --- | --- | --- |
|  | Parameter value in sensitivity analysis | NH Black males | NH White males | NH Black females | NH White females | NH Black males | NH White males | NH Black females | NH White females | NH Black males | NH White males | NH Black females | NH White females |
| Basecase |  | 20 | 20 | 20 | 20 | 261 | 693 | 634 | 1060 | 43% | 41% | 40% | 38% |
| Sensitivity (basecase: 91.5%) | 100% | 20 | 20 | 20 | 20 | 264 | 707 | 646 | 1081 | 47% | 45% | 45% | 41% |
|  | 80% | 20 | 20 | 20 | 20 | 266 | 703 | 646 | 1067 | 37% | 37% | 36% | 34% |
|  | 70% | 20 | 20 | 20 | 20 | 267 | 702 | 645 | 1059 | 33% | 32% | 31% | 30% |
| Eradication rate (basecase: 80%) | 100% | 20 | 20 | 20 | 20 | 213 | 566 | 518 | 860 | 53% | 52% | 51% | 48% |
|  | 90% | 20 | 20 | 20 | 20 | 236 | 630 | 573 | 955 | 48% | 47% | 46% | 43% |
|  | 70% | 20 | 20 | 20 | 20 | 303 | 805 | 738 | 1228 | 37% | 37% | 36% | 33% |
|  | 60% | 20 | 20 | 20 | 20 | 354 | 944 | 870 | 1441 | 32% | 31% | 30% | 28% |
| Effect of *H. pylori* eradication on AG progression (basecase: x3.6) | no effect | 20 | 20 | 20 | 20 | 268 | 716 | 657 | 1102 | 42% | 41% | 40% | 37% |
|  | 1.5x calibrated value | 20 | 20 | 20 | 20 | 265 | 708 | 646 | 1083 | 43% | 41% | 41% | 38% |
| Effect of *H. pylori* eradication on IM progression (basecase x1.12) | no effect | 20 | 20 | 20 | 20 | 267 | 716 | 655 | 1100 | 43% | 41% | 40% | 37% |
|  | 1.5x calibrated value | 20 | 20 | 20 | 20 | 262 | 699 | 631 | 1042 | 43% | 42% | 42% | 39% |

**Suppl. Table 7.** Sensitivity analyses on the screen-and-treat parameters of MISCAN-gastric. The optimal age to screen-and-treat did not change in any analysis. NH, non-Hispanic; *H. pylori, Helicobacter pylori*.

# References

1. Habbema JDF, Van Oortmarssen GJ, Lubbe JTN, Van der Maas PJ. The MISCAN simulation program for the evaluation of screening for disease. Computer methods and programs in biomedicine. 1985;20(1):79-93.

2. Puttelaar van den R. Advancing Colorectal Cancer Screening: Challenges and Innovations. Rotterdam: Erasmus University; 2025.

3. Van Hees F, Zauber AG, Van Veldhuizen H, Heijnen M-LA, Penning C, de Koning HJ, et al. The value of models in informing resource allocation in colorectal cancer screening: the case of the Netherlands. Gut. 2015;64(12):1985-97.

4. Irzaldy A, Gvamichava R, Beruchashvili T, Sturua L, van Ravesteyn NT, de Koning HJ, Heijnsdijk EAM. Breast Cancer Screening in Georgia: Choosing the Most Optimal and Cost-Effective Strategy. Value in Health Regional Issues. 2024;39:66-73.

5. Heijnsdijk EAM, Wever EM, Auvinen A, Hugosson J, Ciatto S, Nelen V, et al. Quality-of-life effects of prostate-specific antigen screening. New England Journal of Medicine. 2012;367(7):595-605.

6. Correa P. Human gastric carcinogenesis: a multistep and multifactorial process—first American Cancer Society award lecture on cancer epidemiology and prevention. Cancer research. 1992;52(24):6735-40.

7. Gupta S, Li D, El Serag HB, Davitkov P, Altayar O, Sultan S, et al. AGA clinical practice guidelines on management of gastric intestinal metaplasia. Gastroenterology. 2020;158(3):693-702.

8. de Kok IMCM, van Rosmalen J, Dillner J, Arbyn M, Sasieni P, Iftner T, van Ballegooijen M. Primary screening for human papillomavirus compared with cytology screening for cervical cancer in European settings: cost effectiveness analysis based on a Dutch microsimulation model. Bmj. 2012;344:e670.

9. van den Broek JJ, van Ravesteyn NT, Heijnsdijk EA, de Koning HJ. Simulating the impact of risk-based screening and treatment on breast cancer outcomes with MISCAN-Fadia. Medical Decision Making. 2018;38(1_suppl):54S-65S.

10. van der Steen A, van Rosmalen J, Kroep S, van Hees F, Steyerberg EW, de Koning HJ, et al. Calibrating parameters for microsimulation disease models: a review and comparison of different goodness-of-fit criteria. Medical Decision Making. 2016;36(5):652-65.

11. Mitchell M. An introduction to genetic algorithms: MIT press; 1998.

12. Helton JC, Davis FJ. Latin hypercube sampling and the propagation of uncertainty in analyses of complex systems. Reliability Engineering & System Safety. 2003;81(1):23-69.

13. Bae J-M, Shin SY, Kim EH. Mean sojourn time of preclinical gastric cancer in Korean men: a retrospective observational study. Journal of Preventive Medicine and Public Health. 2014;47(4):201.

14. Morais S, Costa A, Albuquerque G, Araújo N, Tsugane S, Hidaka A, et al. “True” Helicobacter pylori infection and non‐cardia gastric cancer: A pooled analysis within the Stomach Cancer Pooling (StoP) Project. Helicobacter. 2022;27(3):e12883.

15. Taylor CS, McMahon MV, Ward ZJ, Alarid-Escudero F, Camargo MC, Laszkowska M, et al. Birth cohort and age-specific trends in global Helicobacter pylori seroprevalence: a scoping review. The Lancet Regional Health–Americas. 2024.

16. Mülder DT, Hahn AI, Huang RJ, Zhou MJ, Blake B, Omofuma O, et al. Prevalence of Gastric Precursor Lesions in Countries with Differential Gastric Cancer Burden: A Systematic Review & Meta-Analysis. Clinical Gastroenterology and Hepatology. 2024.

17. Huang RJ, Ende AR, Singla A, Higa JT, Choi AY, Lee AB, et al. Prevalence, risk factors, and surveillance patterns for gastric intestinal metaplasia among patients undergoing upper endoscopy with biopsy. Gastrointestinal endoscopy. 2020;91(1):70-7. e1.

18. Genta RM, Sonnenberg A. Characteristics of the gastric mucosa in patients with intestinal metaplasia. The American Journal of Surgical Pathology. 2015;39(5):700-4.

19. Laszkowska M, Truong H, Faye AS, Kim J, Tan SX, Lim F, et al. Prevalence of extensive and limited gastric intestinal metaplasia and progression to dysplasia and gastric cancer. Digestive diseases and sciences. 2022:1-9.

20. Zhu F, Zhang X, Li P, Zhu Y. Effect of Helicobacter pylori eradication on gastric precancerous lesions: A systematic review and meta‐analysis. Helicobacter. 2023;28(6):e13013.

21. You W-c, Brown LM, Zhang L, Li J-y, Jin M-l, Chang Y-s, et al. Randomized double-blind factorial trial of three treatments to reduce the prevalence of precancerous gastric lesions. Journal of the National Cancer Institute. 2006;98(14):974-83.
